# Association of SSR markers with functional traits from heat stress in diverse tall fescue accessions

**DOI:** 10.1186/s12870-015-0494-5

**Published:** 2015-05-10

**Authors:** Xiaoyan Sun, Zhimin Du, Jin Ren, Erick Amombo, Tao Hu, Jinmin Fu

**Affiliations:** Key Laboratory of Plant Germplasm Enhancement and Specialty Agriculture, Wuhan Botanical Garden, Chinese Academy of Science, Wuhan, 430074 Hubei P.R. China; The Key Laboratory of Horticultural Plant Genetic and Improvement of Jiangxi, Institute of Biology and Resources, Jiangxi Academy of Sciences, Nanchang, 330096 China

**Keywords:** Association mapping, Tall fescue, Population structure, High-temperature stress, Functional traits

## Abstract

**Background:**

Heat stress is a critical threat to tall fescue in transitional and warm climate zones. Identification of association between molecular markers and heat tolerance-related functional traits would promote the efficient selection of heat tolerant tall fescue cultivars. Association analysis of heat tolerance-related traits was conducted in 100 diverse tall fescue accessions consisting of 93 natural genotypes originating from 33 countries and 7 turf-type commercial cultivars.

**Results:**

The panel displayed significant genetic variations in growth rate (GR), turfgrass quality (TQ), survival rate (SR), chlorophyll content (CHL) and evapotranspiration rate (ET) in greenhouse and growth chamber trials. Two subpopulations were detected in the panel of accessions by 1010 SSR alleles with 90 SSR markers, but no obvious relative kinship was observed. 97 and 67 marker alleles associated with heat tolerance-related traits were identified in greenhouse trial and growth chamber trial (*P* < 0.01) using mix linear model, respectively. Due to different experimental conditions of the two trials, 2 SSR marker alleles associated with GR and ET were simultaneously identified at *P* < 0.01 level in two trials in response to heat stress.

**Conclusion:**

High-temperature induced great variations of functional traits in tall fescue accessions. And the identified marker alleles associated with functional traits could provide important information about heat tolerance genetic pathways, and be used for molecular assisted breeding to enhance tall fescue performance under heat stress.

**Electronic supplementary material:**

The online version of this article (doi:10.1186/s12870-015-0494-5) contains supplementary material, which is available to authorized users.

## Background

Tall fescue (*Festuca arundinacea* Schreb.) is a major cool-season grass species from the family Poaceae. Native to Northern Europe, North Africa, Middle East, Central Asia, and Siberia, tall fescue is most widely utilized as forage and turfgrass attributed to its adaptability, yield, persistence, and other ecosystem services such as soil improvement, recreation, protection, and carbon sequestration. Tall fescue is a self-incompatible allohexaploid (2n = 6x = 42) out-crossing species containing three genomes (P, G1, and G2) with a genome size of approximately 5.27-5.83 × 10^6^ kb [1].

Heat stress limits the growth and development of tall fescue in transitional and warm climatic regions. High summer temperature of 30 to 35°C could constrain growth, reduce turf quality, induce leaf withering, and inhibit photosynthesis [2], which would pose severe effects on global climate change. While effective agronomic measures, including heat acclimation, soil temperature reduction, and growth regulators application, could enhance heat tolerance of tall fescue. Heat tolerant cultivars would be key alternative in alleviation of the negative influences of abiotic stress on plant breeding programs [3]. However, plant heat tolerance is a complex quantitative trait, involving multiple regulatory mechanisms, signal transduction pathways, and metabolic pathways. Therefore, a study on genetic and molecular basis for heat tolerance in plants would be necessary. Detailed study in plant physiological responses to heat stress and identification of molecular markers linked to heat tolerance would enhance the efficiency of traditional breeding programs to developing heat tolerant cultivars.

The quantitative inheritances of heat tolerance and interaction between gene expression and environment make challenges to our knowledge of genetic basis of heat tolerant traits of plant. During last two decades, molecular marker has applied to insight into complex traits in plant. Many studies on quantitative trait locus (QTLs) mapping have been conducted to dissect numerous vital agronomical and morphological traits under abiotic stress. The results have improved the efficiency of conventional crop breeding via marker-assisted selection (MAS) in some crop species e.g. rice, maize, barley, soybean, and chickpea [4-8]. However, many linkage mapping based on QTLs studies presented modest and unreliable results due to several factors. First, mapping-based cloning of QTL is time-consuming and costly for construction of populations. Secondly, the restricted number of recombination events per chromosome during mapping population development limits the resolution of genetic map [9]. In addition, QTL mapping could not exploit the extensive genetic variation of natural germplasm resources. On the contrary, association mapping could exploit all recombination events and mutations including historical and evolutionary recombination in natural populations with unobserved ancestry [10]. Association mapping has been widely applied to explore the genetic basis of complex quantitative traits in plant species, and reported under favorable conditions like drought [11-14]. For example, a candidate gene, *ZmDREB2.7* associated with drought stress, was identified to be effective in imparting plant tolerance to drought stress in maize [13]. In turfgrass species, a few studies on association mapping have been carried out involving flowering time, leaf length, submergence tolerance, salinity tolerance, and drought tolerance in perennial ryegrass [15-17]. Four single nucleotide polymorphisms from *LpLEA3*, *LpFsSOD*, and *Cu-ZnSOD* have been associated with drought tolerance traits in diverse perennial ryegrass accessions [14]. However, there was limited information on the association between marker genes and heat tolerance of plants [8].

Simple sequence repeats (SSRs) or microsatellites are widely distributed in all eukaryotic genomes. They are powerful tools for dissecting cultivar fingerprinting, genetic diversity assessment, evolutionary study, linkage map construction, and marker assisted breeding [18-20]. Alternatively, the SSR markers were developed for allohexaploid tall fescue, an out-crossing species with high intra-specific polymorphism, utilized for genomic mapping, identification of variety, population genetic analysis and diversity evaluation of germplasm [21-25]. Recently, SSR markers have been applied in trait and marker association of plants, such as kernel size and milling quality in wheat [26], oil, starch, and protein concentration in maize [27], submergence tolerance in perennial ryegrass [17]. However, the application of association mapping in detecting links between markers with functional traits such as heat tolerance in tall fescue is undocumented.

The objective of this study was to identify marker-trait associations for phenotypic and physiological traits under heat conditions. It was hypothesized that tall fescue accessions had high diversity in high temperature response and the population structure would influence individual functional traits associated with heat tolerance. A set of 100 diverse tall fescue accessions originating from different geographical regions was grown in two heat environmental conditions in the greenhouse and controlled growth chambers. The population structure, relative pairwise kinship, and marker-trait association (MTA) by mixed linear model were statistically analyzed based on SSR markers.

## Results

### Heat stress effects and functional traits variation

In tall fescue heat stress imposed leaf yellowing and wilting, limited plant growth, and even death. Turfgrass quality (TQ), survival rate (SR), chlorophyll content (CHL), and growth rate (GR) decreased with prolonged heat stress in both trials, but the severity of decline varied with accession and duration. Significant accession and treatment time effects under heat stress were observed on GR, TQ, CHL, and SR in both trials (Table 1). However, no significant time effect for evapotranspiration rate (ET) in growth chamber trial was detected. There was also no significant interaction effect for functional traits between grass accessions and treatment time.

**Table 1:** **Mean squares of variance for evapotranspiration rate** (**ET**), **growth rate** (**GR**), **turf quality (TQ), chlorophyll content (CHL), and survival rate (SR) of 100 tall fescue accessions on different times of heat treatment**

**Sources**	**Greenhouse trial**					**Growth chamber trial**				
	**ET**	**GR**	**TQ**	**CHL**	**SR**	**ET**	**GR**	**TQ**	**CHL**	**SR**
**Accession (A)**	1725.3**	1828.9**	755.5**	519.5**	578.7**	10693.3**	17087.7**	1313.1**	2127.2**	1135.2**
**Time (T)**	2229801.4**	189804.5**	128139.4**	30165.8**	57682.3**	7400.5^ns^	299505.0**	73169.6**	29394.0**	96450.8**
A*T	483.5^ns^	453.8^ns^	111.3^ns^	166.0 ^ns^	90.2^ns^	2214.4^ns^	3555.4^ns^	168.3^ns^	731.3^ns^	161.2^ns^

**mean significant at *P < 0.01*, and ^ns^mean no significant.

With prolonged heat stress at 1-3 weeks, the mean, maximum, and minimum values decreased in two trials (Table 2). Under heat stress, the average growth rate decreased from 0.24 g d^-1^ at initial time to 0.05 g d^-1^ at 14 WOT, turfgrass quality reduced from 6.55 to 2.56, survival rate decreased from 99.65% to 46.66%, chlorophyll content decreased from 2.35 mg g^-1^ FW to 1.47 mg g^-1^ FW, and evapotranspiration rate decreased from 61.55 g d^-1^ to 10.64 g d^-1^, respectively in greenhouse trial. Most of the functional traits decreased except for ET, which increased after one week of stress treatment, and then drastically dropped. In growth chamber trial, all functional traits displayed similar trend, whereby the average GR dropped from 0.11 g d^-1^ at initial time to 0.03 g d^-1^ at two WOT, TQ from 7.50 to 3.01, SR from 99.75% to 52.50%, CHLT from 2.03 mg g^-1^ FW to 1.77 mg g^-1^ FW, and ET from 22.82 g d^-1^ to 18.93 g d^-1^, respectively. After two weeks, heat stress significantly reduced GR by 79.17% in greenhouse and 72.73% in growth chamber trials compared with their relative controls (the time before heat stress). The decline levels of TQ, SR, and ET were lower than that of GR.

**Table 2:** **Descriptive statistics for growth rate (GR), turfgrass quality (TQ), survival rate (SR), evapotranspiration rate (ET), chlorophyll content (CHL) under heat stress in two trials**

**Trait**	**Greenhouse trial**				**Growth chamber trial**			
	**Minimum**	**Maximum**	**Mean**	**Std.**	**Minimum**	**Maximum**	**Mean**	**Std.**
GR-1^a^ (g/d)	0.04	15.29	0.24	0.8	0.03	0.25	0.11	0.04
GR-2^b^ (g/d)	0.01	0.41	0.11	0.05	0.01	0.19	0.06	0.03
GR-3^c^ (g/d)	0.00	0.24	0.05	0.03	0.00	0.07	0.03	0.02
TQ-1^a^	3.00	8.50	6.55	0.93	6.00	9.00	7.50	0.58
TQ-2^b^	2.00	7.00	4.72	1.14	2.33	7.67	5.54	1.15
TQ-3^c^	0.00	6.00	2.56	0.88	1.00	6.17	3.91	1.24
CHL-1^a^ ( mg.g^-1^.Fw )	1.39	6.22	2.35	0.55	1.32	3.20	2.03	0.41
CHL-2^b^ ( mg.g^-1^.Fw )	0.70	3.08	1.76	0.44	1.26	3.05	2.05	0.44
CHL-3^c^ (mg.g^-1^.Fw)	0.09	3.11	1.47	0.46	0.28	3.43	1.77	0.55
SR-1^a^ (%)	90.00	100.00	99.65	1.33	98.33	100.00	99.75	0.60
SR-2^b^ (%)	35.00	95.00	71.92	9.27	36.67	95.00	77.78	13.07
SR-3^c^ (%)	5.00	75.00	46.66	16.49	6.67	86.67	52.5	16.08
ET-1^a^ (g/d)	23.50	97.00	61.55	11.38	8.37	47.12	22.82	6.93
ET-2^b^ (g/d)	30.00	142.00	84.84	10.99	6.96	49.66	21.17	7.70
ET-3^c^ (g/d)	1.00	48.00	10.64	5.80	4.13	33.17	18.93	6.62

1^a^ sampling time before heat treatment.

2^b^ sampling time after 7d of heat treatment.

3^c^ sampling time after 14d of heat treatment.

Significant correlations between survival rate with evapotranspiration rate, turfgrass quality, and turfgrass quality with evapotranspiration rate were found at two time of heat stress that the values had been standard to relative control in greenhouse trial, and relative values of SR of two weeks under heat stress had significant relationship with chlorophyll content (Table 3). Meanwhile, there were significant correlations between turfgrass quality with GR, CHL, ET and SR in growth chamber trail. There was significant correlation between CHL and SR, ET and GR, however there was no relationship between ET and SR in growth chamber trail (Table 4). High correlations were identified for all functional traits between the two sample times under heat stress in both trials, with the highest correlation for ET (*r* = 0.86, *P* < 0.01) in greenhouse trial, and SR (*r* = 0.768, *P* < 0.01) in growth chamber trial.

**Table 3:** **Pearson correlations coefficients among functional traits of different time in greenhouse trial**

	**GR-1** ^**a**^	**GR-2** ^**b**^	**TQ-1** ^**a**^	**TQ-2** ^**b**^	**ET-1** ^**a**^	**ET-2** ^**b**^	**CHL-1** ^**a**^	**CHL-2** ^**b**^	**SR-1** ^**a**^	**SR-2** ^**b**^
**GR-1** ^**a**^	1									
**GR-2** ^**b**^	0.684^**^	1								
**TQ-1** ^**a**^	-0.129	-0.209^*^	1							
**TQ-2** ^**b**^	-0.002	-0.085	0.698^**^	1						
**ET-1** ^**a**^	0.060	-0.025	0.231^*^	0.276^**^	1					
**ET-2** ^**b**^	0.059	-0.006	0.280^**^	0.283^**^	0.850^**^	1				
**CHL-1** ^**a**^	-0.100	-0.080	0.097	0.141	-0.130	-0.086	1			
**CHL-2** ^**b**^	-0.328^**^	-0.361^**^	0.278^**^	0.155	-0.030	0.015	0.444^**^	1		
**SR-1** ^**a**^	0.045	-0.011	0.731^**^	0.568^**^	0.315^**^	0.322^**^	0.146	0.104	1	
**SR-2** ^**b**^	-0.031	-0.197^*^	0.602^**^	0.699^**^	0.333^**^	0.313^**^	0.197^*^	0.265^**^	0.644^**^	1

*significant at *P <* 0.05, **significant at *P <* 0.01.

1^a^ the value at 7 d of heat stress relative the initial value before heat stress.

2^b^ the reduction value at 14d of heat stress relative the initial value.

Abbreviations: *TQ*-turf quality, *ET*- evapotranspiration rate, Chl-chlorophyll content. *SR*-survival rate, *GR*- Growth rate.

**Table 4:** **Pearson correlations coefficients among functional traits of different time in growth chambers trial**

	**GR-1** ^**a**^	**GR-2** ^**b**^	**TQ-1** ^**a**^	**TQ-2** ^**b**^	**ET-1** ^**a**^	**ET-2** ^**b**^	**CHL-1** ^**a**^	**CHL-2** ^**b**^	**SR-1** ^**a**^	**SR-2** ^**b**^
**GR-1** ^**a**^	1									
**GR-2** ^**b**^	0.608^**^	1								
**TQ-1** ^**a**^	0.316^**^	0.327^**^	1							
**TQ-2** ^**b**^	0.243^*^	0.444^**^	0.686^**^	1						
**ET-1** ^**a**^	0.184	0.262^**^	0.251^*^	0.323^**^	1					
**ET-2** ^**b**^	0.085	0.315^**^	0.159	0.417^**^	0.688^**^	1				
**CHL-1** ^**a**^	-0.036	0.109	0.119	0.181	0.036	0.054	1			
**CHL-2** ^**b**^	0.031	0.230^*^	0.361^**^	0.403^**^	0.015	0.121	0.525^**^	1		
**SR-1** ^**a**^	0.059	0.246^*^	0.373^**^	0.365^**^	-0.044	-0.016	0.264^**^	0.587^**^	1	
**SR-2** ^**b**^	-0.022	0.193	0.300^**^	0.326^**^	-0.022	0.001	0.182	0.606^**^	0.768^**^	1

*significant at *P < 0.05*, **significant at *P < 0.01*.

1^a^ the value at 7 d of heat stress relative the initial value before heat stress.

2^b^ the reduction value at 14d of heat stress relative the initial value.

Abbreviations: *TQ*-turf quality, *ET*- evapotranspiration rate, Chl-chlorophyll content. *SR*-survival rate, *GR*- Growth rate

### Population structure, relative kinship

A total of 1010 SSR alleles were amplified from 90 SSR markers by genotyping 100 tall fescue accessions (Additional file 1 Table S1). The allele numbers of SSR marker varied from 3 to 27 alleles per marker with an average of 11.22 alleles per locus. For the co-dominant SSR marker transit to dominant marker in this study, the genetic diversity of the 100 tall fescue accessions was at a relative lower level, in which average of *Nei*’s genetic diversity was 0.255, and average of polymorphism information content was 0.211.

**Figure 1:**
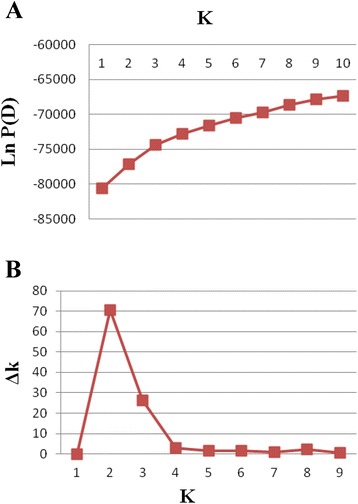
Calculation of true K of tall fescue accessions and **(A)** Evolution of the average logarithm probability of the data likelihoods (LnP(D)) for tall fescue genotypes; **(B)** Magnitude of Δk for each K value according to Evanno *et al.* [58].

**Figure 2:**
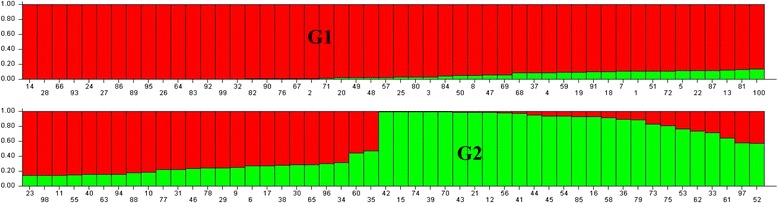
Population structure analysis of 100 tall fescue accessions. Numbers on the x-axis indicate the accession and those on the y-axis show the group membership.

According to STRUCTURE analysis results based on Bayesian clustering approach model, a significant population structure was detected among the 100 accessions. The results were consistent with those from the preliminary runs, in which the average probability of the data likelihoods for the population structure in the panel of accessions were increased following the increase of K (Figure 1A). Therefore, the likely number of subpopulations was identified using the Delta method. The optimal number of groups was determined by the maximum likelihood, and k was set at 2 implying two structural groups (G1 and G2) were identified in the panel (Figure 1B). The population structure matrix (Q) identified at k = 2 was applied to define the membership probability for assigning accessions to subpopulation when the value was >0.7 (Additional file 2 Table S2). However, a few of wild accessions were obscure, such as 4 (Q1 = 0.542), 55 (Q2 = 0.578), 62 (Q1 = 0.553), and 96 (Q2 = 0.583). Most of value of accessions (80%) were >0.8. G1 that was the most diverse group contained 74 accessions of mixed origins, including all commercial cultivars (8 accessions) and the majority of wild accessions form European (25/32), U.S. (28/30), Asia (8/20), and South America (3/3) (Figure 2). G2 contained 26 accessions that mainly collected majorly from North Africa (5/6, Tunisia, Algeria, and Morocco), Asia (12/20, China, Iran, Turkey, Israel), European (7/32).

**Figure 3:**
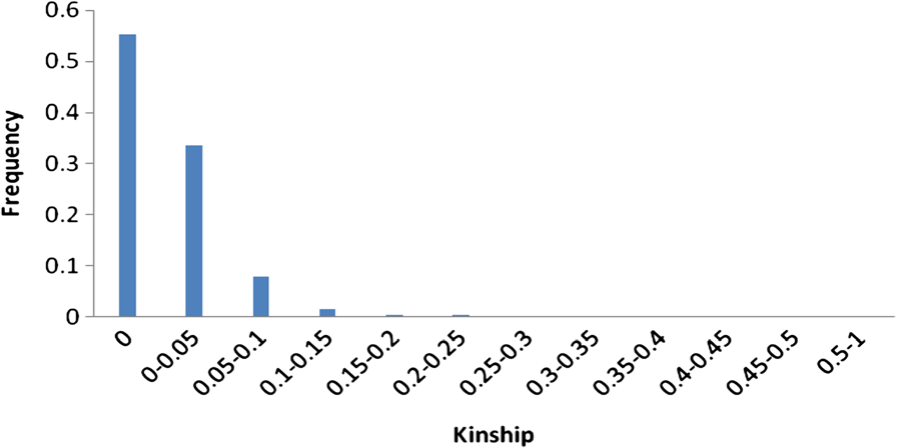
Distribution of pair-wise relative kinship estimates between 100 tall fescue accessions.

There was no obvious kinship (K) that detected based on 90 SSR markers in the panel of populations (Figure 3). More than 55.3% of the pair-wise kinship estimates were zero while approximately 89% of estimates were between 0 and 0.05. Less than 5% of estimates were >0.1, indicating that the familial relationships minimum among samples, and would not cause further complexity in association analysis.

### Association analysis and evaluation of association model

**Figure 4:**
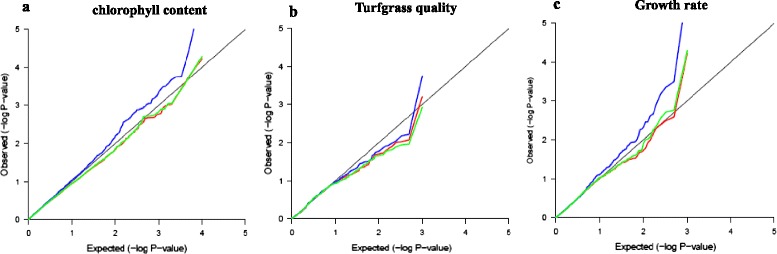
Quantile-quantile plots of estimated –log10 (P) from association analysis using three models in three traits: **a** turfgrass quality, **b** growth rate, **c** leaf chlorophyll content. The *black line* is the expected line under the null distribution. The blue line represents the observed P values using GLM with Q model; the r*ed line* represents the observed P values using MLM model with K; the *yellow line* represents the observed P values using MLM model with Q and K.

Combined with all SSR alleles and three traits including turfgrass quality, growth rate and leaf chlorophyll content in growth chamber trial, associations were performed to detect the effects of *Q* and *K* for controlling false associations. Owing to the complexity and population structure in out panel, the simple model that overlooked *Q* and *K* was not performed. For any trait, the *P* values from the three models were close to the expected *P* value (Figure 4). However, the model of *Q* showed a different distribution with the other models for turfgrass quality and chlorophyll content. On the other hand, the *K* and *Q + K* model displayed similar distribution of *P* values, and the identified associations (*P* < 0.01) showed the high similarity in both models (Additional file 3 Table S3). The more stringent model was performed, and the less spurious associations were identified. So the results from *Q + K* model by MLM would be showed and discussed.

### Marker allele-trait associations

In MLM model with *Q* and *K*, a total of 97 SSR alleles were associated with five heat-relative traits at two time points (*P* < 0.01) in greenhouse trial, while that in growth chamber trial resulted in 67 SSR loci that were strongly associated with the 5 traits (*P* < 0.01) (Table 5, and Additional file 4 Table S4). In greenhouse trial, 15 alleles of marker NAF057 that amplified 22 alleles showed the association with ET at two time points by using *Q + K* model. The similar results also occurred in marker NFA87 associated with GR-1, marker NFA155 related with TQ-1 in growth chamber trial. Moreover, many marker alleles could be associated with a functional trait, and one marker allele was associated with more than one trait. For example, SSR marker alleles (NAF036-194, NAF013-250, NAFG17-136, NAFG023-207, and NAF138-211) were associated with SR and TQ at two sampling times under heat stress in greenhouse trial. Comparing with the same association alleles in two trials, only 2 marker alleles showed similar associations with traits (Table 6). NFA87-418 that located the linkage group 3B was associated with GR-2, and NFA91-152 was associated with ET-2 in both trials by MLM analysis.

**Table 5:** **Significant marker-trait associations identified for different traits by Q + K model of MLM**

**Trial**	**Trait**	**Growth chamber trial**			**Greenhouse trial**		
		**Number of markers**	**P-value range**	**Phenotypic variation (%)**	**Number of markers**	**P-value range**	**Phenotypic variation (%)**
**Greenhouse**	**GR-1**	22	3.93 × 10^-4^-0.0096	7.23-17.38	11	3.06 × 10^-4^-0.0093	7.09-13.93
	**GR-2**	7	6.31 × 10^-5^-0.0069	7.77-21.86	2	0.0038-0.0057	13.22-14.34
	**TQ-1**	8	0.0030-0.0096	7.11-12.62	8	1.57 × 10^-4^-0.0093	7.03-14.09
	**TQ-2**	2	7.51 × 10^-4^-0.0087	7.20-12.17	4	0.0030-0.0075	9.06-10.10
	**CHLT-1**	5	8.59 × 10^-4^-0.0099	7.06-15.51	17	0.0025-0.0094	8.80-12.99
	**CHLT-2**	6	0.0024-0.0078	8.14-13.94	4	0.0049-0.0073	10.38-11.27
	**SR-1**	2	0.0011-0.0073	8.77-17.22	8	8.09 × 10^-4^-0.0090	7.15-15.46
	**SR-2**	4	0.0028-0.0065	8.90-13.92	3	0.0089-0.0099	7.66-10.99
	**ET-1**	5	0.0039-0.0057	10.49-13.52	20	7.29 × 10^-4^-0.0089	7.25-15.17
	**ET-2**	6	0.0028-0.0085	7.36-12.48	20	2.86 × 10^-4^-0.0092	7.17-17.06

*Abbreviations:* *TQ*-turf quality, *ET*- evapotranspiration rate, Chl-chlorophyll content. *SR*-survival rate, *GR*- Growth rate.

**Table 6:** **Same marker allele-trait associations with percentage of functional traits after 7 and 14 d of heat stress relative to initial condition of tall fescue accessions for both trials by Q + K model at** ***P*** **< 0.01**

**Marker allels**	**LG** ^**a**^	**Greenhouse trial**				**Growth chamber**			
		**Trait**	**marker_F**	**marker_p**	**markerR2**	**Trait**	**marker_F**	**marker_p**	**markerR2**
NFA87-418	3B	GR-2	5.9196	0.0038	0.1434	GR-2	5.9196	1.88 E-04	0.1916
NFA91-152	NA	ET-2	4.9308	0.0092	0.1001	ET-2	5.1261	0.0077	0.1046

LG^a^ mean the locus of linkage groups of genetic linkage map of tall fescue according to Sara *et al.* [21].

Abbreviations: *ET*- evapotranspiration rate, *GR*- Growth rate.

## Discussion

### Heat responses of tall fescue

Heat stress is a major factor that limits growth of cool-season turfgrass on a global scale. Turfgrass survive under high temperature through tolerance or escape mechanisms, which involve many phenotypic and physiological characteristics including growth-restricted, higher photosynthesis rate, stay-green, cell membrane thermal stability, and earliness [28]. High temperature decreased turf quality, caused leaf water deficiency and yellowing, constrained growth, and reduced photosynthesis. So, leaf wilting, turfgrass quality, growth rate, evorpotranspiration rate, and chlorophyll content provided convenient and more efficient measurements for studying turfgrass responding mechanism under unfavorable conditions, which have been intensively applied for screening heat-tolerant germplasm of turfgrass [29-31].

In our trials, tall fescue accessions under heat stress exhibited varying degree of negative effects based on ANOVA analysis. Relatively low TQ, high leaf wilting, reduced CHL and severe water loss characteristics presented the damage level of heat stress of tall fescue. Large variations in these functional traits of accessions from different geographic locations and significant correlations between functional traits would provide the potential for selecting heat tolerant accessions and evaluating reliable SSR marker by association analysis between marker and functional traits. However, heat tolerance mechanisms of tall fescue would be different in the two trials. In growth chamber trial, heat tolerant accessions maintained relatively high growth rate and good turfgrass quality. Simultaneously, heat-sensitive accessions presented lost water rapidly, curled leaves and even died. Meanwhile, in the greenhouse trial, heat tolerant accessions maintained good turfgrass quality and appearance by restraining growth. Similarly heat-sensitive accessions experienced yellowing of leaves and withering. The probable explanation for variations in tolerance mechanisms under heat stress were due to the different stress conditions including soil properties and temperature [32]. In the greenhouse trial the roots temperature was buffered because of properties of soil. But flasks with roots were directly exposed to heat stress in the chamber trial due to utilizing the nutrition solution, which made the turfgrass in flask to be more sensitive to high temperature than in the greenhouse trial. In addition, both trials displayed highly significant correlations in most of the functional traits. This indicated that heat tolerant traits had mutual influence, and these traits could provide adequate parameters for evaluating the heat tolerance in the field. Tall fescue accessions from different collection areas indicated diversity in phenotypic and physiological characteristics [33]. However the trend and level of heat damage of the accessions were roughly consistent in two time points. Therefore, heat tolerant tall fescue accessions would be effectively selected according the phenotypic traits when heat stress conducted early days.

### Population structure

Tall fescue accessions native to Europe and North Africa, were introduced to North and South America in the late 1800s. They eventually became a prominent forage grass in 1940s in the United States where many commercial cultivars were produced through selective breeding [34]. Tall fescue samples were collected from more than 40 cities representing diverse geographical origins. So in view of the geographical origins, local adaptation, and breeding history of genotypes in association mapping panel, the non-independent samples would often encompass both population structure and familiar relatedness [35,36]. In our study, the Bayesian clustering approach model based analysis divided the panel of samples into two sub-populations. The most of the accessions from European, North America, and all commercial cultivars were separated into the main subpopulation. The wild accessions from North Africa and Asia were separated into the second subpopulation. The division rule cannot be simple explained geographically due to overlapping of several accessions from the same region (European and Asia) in two groups, which indicated regional breeding objectives, the probable different evolutionary paths and methods of ecological adaptation in morphology and agronomic characteristics of ecogeographic races would be considered [37,38].

Presence of population structure could make some allele frequencies significantly differ between subpopulations, which would lead to spurious association (false positives) of markers with traits [39]. Flint-Garcia et al. [40] presented that 33 to 35% of variation of phenotypic traits about flowering time in a diverse maize panel would be attributed to population structure. Therefore, if subpopulation structure is not taken into account, spurious associations may be identified at other loci that were differentially distributed among subpopulations. Moreover, spurious associations cannot be controlled entirely by GLM model. This is because the *Q* matrix can only carry a rough dissection of population differentiation. Therefore, a unified mixed-model approach for association mapping that incorporates the pairwise kinship (*K* matrix) and *Q* matrix to correct multiple levels of relatedness have been developed. This would be a powerful approach for improving accuracy of association in many cases [40,41]. Kang [42] demonstrated that the distribution of *P* values ideally should follow a uniform distribution with less deviation from the expected *P* value. In our panel, SSR marker-trait associations were performed for three traits using the *Q*, *K*, and *Q + K* models, and all the three showed a good fit for *P* values. However, the models showed the different effects of controlling the population structure for different traits. *K* model was more superior to the *Q* model, but similar to the *Q + K* model. This is consistent with some previous studies [41,43]. The *K* matrix could capture the relatedness between each possible pair of individual in panel. The *Q* matrix considers a few axes only [44]. Consequently, no vivid familiar relatedness (from the recent co-ancestry) has been detected in the panel. Therefore a model that would test for complex quantitative traits would be necessary for improving the accuracy of association.

### Marker allelic effects on functional traits

Little is known about the association of SSR loci with heat tolerance related traits in plant species. In our study 97 marker-trait associations (MTAs) in greenhouse trial and 67 MTAs were identified for five heat tolerant traits at two time points (*P* < 0.01). A total of 13 MTAs in greenhouse trial and 29 MTAs in growth chamber trial were identified in present study for growth rate. High temperature would affect pollen viability, fertilization and seed development leading to yield losses. A large number of MTAs for yield and yield related traits under unfavorable conditions were reported in many crop species [7,8,13]. Furthermore, most of marker NAF057 amplifying 22 alleles were associated with ET in greenhouse trial, which implied the marker may be linked with a crucial gene that is necessary for regulating water loss and transpiration cooling under heat stress [45]. Similar results were observed between survival rate and turfgrass quality in greenhouse trial, suggesting survival rate, turfgrass quality and evapotranspiration rate are vital functional traits reflecting heat tolerance of tall fescue and might be regulated by genetically linked homologous genes. Therefore, these associated markers and identified genotypes with favorable alleles can be deployed after validation for molecular marker breeding to develop heat tolerant in tall fescue.

It is interesting to found that many marker alleles presented significant association with single trait, or associations with more than one trait. For instance, 5 associations were associated with SR and TQ in greenhouse trial, which would be considered to be pleiotropic or co-localized MTAs [8]. These co-localized or pleiotropic associations may be beneficial to detect some important genomic regions or genes for heat tolerance related traits. Furthermore, the markers associated with more than one trait may be made effectively use of improving more than one trait by marker assisted selection.

For screening heat tolerant accessions by phenotypic and physiological traits, our experimental population is relative small, which influence the power of association analysis. Yan et al. [46] showed that association study with a set of 500 individuals would supplyan 80% probability of detecting a gene that explains 3% or more of the phenotypic variation, and increasing the number of population could be more substantial effect on the power of MTAs than increasing the density of markers in genome-wide association (GWS). More reliable markers could be identified for developing elite heat tolerant tall fescue cultivars through marker assisted selection under various conditions: first if higher density DNA polymorphism databases would have been evenly distributed in all genome chromosomes. And secondly larger mapping populations and phenotypic traits under more sites of heat stress would have been used for association mapping.

A large challenge for association analysis for complex quantitative traits in plant is large number of loci identified with small effects in some plant species such as barley, maize, wheat and rice. In our study by MLM analysis, the explained variation (Marker R^2^) for the identified associations were low to modest, ranging from 7.03% (turfgrass quality)-19.21% (turfgrass quality) in greenhouse trial, and 7.06% (leaf chlorophyll content) -21.86% (growth rate) in growth chamber trial, respectively. The explained variation by marker-trait associations (MTAs) for abiotic stress related traits in association analysis of plant species is changeable. Thudi et al. (2014) used DArT, SNP, and SSR markers to study 300 accessions of chickpea for drought tolerance related root traits, heat tolerance, yield and yield component traits cross 6 environments, and showed that phenotypic variance explained of MTAs ranged from low (4.14%) to very high (96.55%). However, Varshney et al.[47] studying a diverse barley panel at a dry and wet location for drought tolerance related traits, found that explained variation for all of identified MTAs was rather low, ranging from 0.1% to 6.7%. Some other studies on GWA analysis in barley also showed that MTAs contributing large phenotypic variation are highly heritable, and MTAs of explained variation >10% seem hard to be identified for the complex quantitative traits like drought tolerance in association analysis [6,48]. Large effect QTL may be due to the inbreeding nature of some species, while out-crossing plants such as tall fescue and maize may have very large number of genes contributing a very small amount to a quantitative trait [46].

Associations identified in our study were not only small, but also little consistent across environments like barley or wheat. In the two trials of our study, there were only two associated SSR alleles were identified in two trials at low threshold, -Log (*P*-value) ≥2.0 by MLM analysis, which showed influence of environment on heat tolerance related traits that would be low heritability. The observed differences of marker-trait associations in both trials may be due to the different experimental conditions of heat stress, including temperature, heat intensity, duration, and matrix cultivated, which lead to the variation of phenotypic, physiologic and biochemical characteristics in response to heat stress, and even trigger different genetic pathways and mechanisms of heat tolerance. In greenhouse trial, the temperature of greenhouse often exceeded 45°C in summer, and reached 50°C at the noon, which restrained growth of tall fescue and caused severe thermal damage. Most tall fescue accessions halted growth, withered rapidly, and the leaves yellowed after 2 or 3 day of heat stress. However, the growth chambers controlled the temperature at 35°C moderate high temperature. The extreme high temperature would induce specific membrane damage, expression of HSP [49], and alteration of activity of enzymes, which was not prevalent at moderate heat stress [50]. Simultaneously, immersing grasses into nutrient solution in growth chamber trial made the roots that are more sensitive to heat stress than leaves to be directly exposed to high temperature and severe damage [51,52]. Therefore, many heat sensitive tall fescue accessions presented dehydration wilting and even death in growth chamber trials. Specifically, many factors resulted in the differences of phenotypic and physical traits when tall fescue accession responded to heat stress, which made a few SSR alleles associated with functional traits to be simultaneously identified. The observation that the majority of the SSR alleles associated with heat tolerant-related traits could only be identified in a specific condition of heat stress indicated that tall fescue is very sensitive to variation of high temperature. The similar conditions had also reported in association analysis for drought tolerance related traits of barley, wheat, maize, and chickpea [7,8,13,46]. So identified markers may be not suitable for direct application in marker-assisted selection (MAS) programme for developing more stable heat tolerant tall fescue varieties or cultivars. Vast studies including complex crosses and QTLs mapping with well chosen parents on the basis of results obtained in our study for verifying effectiveness of marker alleles are necessary for breeding heat tolerance cultivars by marker assisted selection.

## Conclusion

In summary, we initial focus on association mapping analysis of heat tolerance-related functional traits in tall fescue. Five quantitative traits GR, TQ, SR, CHL and ET showed high diversity and significant mutual correlations in response to heat stress in tall fescue. Two subpopulations were detected in the panel of accessions, but no obvious relative kinship was observed. But for any trait, the K model controlling relative kinship showed the similar distribution of P value and associations with Q + K model that controlling both population structure and relative kinship in our study. So model testing is necessary to reduce the spurious associations. By mixed linear model (Q + K) as the best model for association analysis, 97 associations in greenhouse trial and 67 associations in chamber trial were identified for five heat tolerant traits at two time points (*P* < 0.01). It is necessary for tall fescue selection breeding because these markers would enhance efficiency of identifying heat tolerant accessions bringing desirable alleles. However, only two SSR alleles associated with GR and ET were identified due to the different environments between two trials. And inadequate samples and limited markers were utilized in our study which might have weakened the reliability and effectiveness of associated SSR markers. Hence, it was necessary to confirm the associated marker locus by genotypes F2 grasses and phenotype F3 progeny, or QTL mapping with a high resolution linkage mapping in the next step. Simultaneously, for identification of more effective markers or genes by association analysis, further research need to focus on selecting candidate genes regulating heat tolerance of tall fescue or developing a large amount of single nucleotide polymorphism for genotyping larger association population.

## Methods

### Plant materials and growth conditions

100 diverse accessions of tall fescue were employed in this study, including 93 accessions obtained from the United States Department of Agriculture-Agricultural Research Service (USDA-ARS) and 7 turf-type commercial cultivars obtained from the seed industry (Table 7). The collection of accessions was based on geographical locations for maximizing genotypic diversity. All accessions were confirmed to be hexaploid by flow cytometry (data not shown). This study was conducted at Wuhan Botanical Garden, Chinese Academy of Science, beginning in 2012. A single seed from each accession was sown in petri dishes with a layer of filter paper soaked in water and kept in dark at 22°C for germination. After one week, the accessions were transplanted into plastic pots (15 cm deep, 11 cm wide) containing a mixture of sand and soil (1:1, v/v) in a greenhouse with temperature ranging from 20°C to 26°C, 1000-1500 μmol photons m^-2^ s^-1^, 14 h photoperiod of natural sunlight, and 76% average relative humidity. Plants were irrigated daily to maintain sufficient water supply conditions, fertilized weekly with half-strength Hoagland’s solution [53], and mowed to 7 cm canopy height once a week. Each accession was propagated through tillers multiple times for genetic uniformity.

**Table 7:** **Origin and grouping information of tall fescue accessions used in this research**

**ID** ^**a**^	**PI number**	**Origin**	**Q** ^**b**^	**ID** ^**a**^	**PI number**	**Origin**	**Q** ^**b**^
1	Justice	Cultivar	1	51	PI 438521	Japan	1
2	PI 527504	France	1	52	PI 442490	Belgium	1
3	PI 531230	USA	1	53	PI 469244	USA	1
4	PI 595072	Romania	1	54	PI 499494	China	1
5	PI 596701	USA	1	55	PI 499495	China	2
6	PI 598491	Netherlands	1	56	PI 174210	Turkey	2
7	PI 598493	Romania	1	57	PI 200339	Israel	2
8	PI 636532	Tunisia	2	58	PI 203728	Uruguay	1
9	PI 636597	USA	1	59	PI 208679	Algeria	2
10	PI 636601	France	1	60	PI 208681	Algeria	2
11	PI 538006	USA	1	61	PI 211032	Afghanistan	1
12	PI 538330	USA	1	62	PI 224975	South Africa	1
13	PI 577082	Yugoslavia	1	63	PI 231563	Portugal	2
14	PI 578717	USA	1	64	PI 234881	Switzerland	2
15	PI 578718	USA	1	65	PI 234883	Switzerland	1
16	PI 578724	USA	1	66	PI 235036	Sweden	1
17	PI 583747	USA	1	67	PI 235125	Netherlands	1
18	PI 583822	USA	1	68	PI 249738	Greece	1
19	PI 655104	USA	2	69	PI 257742	Sweden	1
20	PI 655112	USA	1	70	PI 269894	Pakistan	1
21	PI 655113	USA	2	71	PI 274617	Poland	2
22	PI 423090	Spain	2	72	PI 283281	UK	1
23	PI 422638	France	1	73	PI 283304	Denmark	1
24	PI 502373	Russian	1	74	PI 311044	Romania	2
25	PI 504538	Greece	1	75	PI 314685	Russian	2
26	PI 577094	Switzerland	1	76	PI 380844	Iran	2
27	PI 505833	Kazakhstan	2	77	PI 388897	Japan	1
28	PI 508603	Argentina	1	78	PI 388898	Japan	1
29	PI 578719	USA	1	79	PI 578714	USA	1
30	PI 512305	Portugal	1	80	PI 601106	USA	1
31	PI 512315	Spain	1	81	PI 601227	USA	1
32	PI 547396	Iran	1	82	PI 601447	USA	1
33	PI 559374	USA	1	83	PI 608024	USA	1
34	PI 561430	USA	1	84	PI 608025	USA	1
35	PI 574522	USA	1	85	PI 619025	China	2
36	PI 577081	Yugoslavia	1	86	PI 632516	USA	1
37	PI 598496	Hungary	1	87	PI 608787	USA	1
38	PI 598574	Kazakhstan	2	88	PI 600739	USA	1
39	PI 598930	Italy	1	89	PI 600801	USA	1
40	PI 598860	Morocco	2	90	3rd Millennium	Cultivar	1
41	PI 610909	Morocco	1	91	Stone wall	Cultivar	1
42	PI 610933	Italy	1	92	Davinci	Cultivar	1
43	PI 610951	Morocco	2	93	Pixie	Cultivar	1
44	Pure Gold	Cultivar	1	94	PI 184041	Yugoslavia	1
45	PI 618971	China	2	95	PI 255874	Poland	1
46	PI 618973	China	2	96	PI 283287	Czechoslovakia	2
47	PI 619005	China	2	97	PI 578713	USA	1
48	PI 440345	Russian	2	98	PI 608808	USA	1
49	PI 423045	Spain	2	99	Grand II	Cultivar	1
50	PI 427127	Chile	1	100	Smirna	Cultivar	1

^a^ID number representing accessions used in this research.

^b^Q identified population structure groups in this research.

### Heat treatment and experimental design

Two trails were conducted. One was processed in the greenhouse in June, 2012, the other in growth chambers repeated in August, September, and October 2012, respectively.

1. Greenhouse trail:

All 100 accessions were transferred into a natural greenhouse in June 8^th^ to July 14^th^, 2012 after growing in the controlled greenhouse for 30 d. The maximum temperatures varied from 39°C to 51°C during 21 d of heat treatment. Each accession had three replications with same genotypes, and all plots were arranged in a completely randomized block design. The greenhouse had a photosynthetically active radiation (PAR) of 1000-2000 μmol s^-1^ m^-2^ of natural sunlight. Grasses were irrigated daily until water could freely drain from the holes under the plots.

2. Growth chamber Experiment

The trial was repeated three times in growth chamber in August, September and October 2012. 100 accessions were transformed into 250 mL Erlenmeyer flask wrapping with aluminum foil, containing half-strength Hoagland’s solution and 0.1 μmol magnesium oxide to provide additional oxygen after 30 d growing in controlled greenhouse. Grasses with 7-10 tillers were sealed with parafilm to prevent water escaping from gaps. Before heat treatment, all flasks of grasses were pre-incubated 10 d. Two growth chambers during experimental period were controlled in 14 photoperiod, 70% ± 10% relative humidity, and approximately average 450 μmol photons m^-2^ s^-1^. Every other day all flasks were exchanged layers and half-strength Hoagland solution added. This trail included an unheated control (25/16°C, day/night) and heat stress (38/30°C, day/ night) treatment sustaining 15 d. The heat treatment was subjected in different chambers for each replication.

### Growth and physiological measurements

Many growth and physiological traits were measured before and after heat stress interval 7 d in two trails, including turf grass quality (TQ), survival rate (SR), leaf chlorophyll content (CHL), evapotranspiration rate (ET) and growth rate (GR) . Turf quality was evaluated visually using a scale of 0 (yellow, brown or dead) to 9 (optimum greenness, uniformity, cover) based on density, texture, turf color, and smoothness. Survival rate was also assessed by visual rate using a ratio between survival canopy and total plant. Every 7 d leaves of pots were cut at 7 cm canopy height, were collected, immediately killed at 105°C 30 min, dried at 70°C in an oven for 72 h. Growth rate was calculated as dry weight per growth day. Evapotranspiration rate was measured by weight loss of the plant plot every 24 h and the relative transproation was normalized according to a method described by Hu et al. [54]. Leaf chlorophyll content was measured using the method described by Hiscox and Israelstam [55].

Data was collected from the non-heat and heat treatment across all accessions of tall fescue from two trails to examine the efficiency and consistency. The percentage of reduction of all traits, calculated as [(control value or initial value -heat value)/ control or initial value] × 100, was used to indicated the grass heat tolerance. The main treatment effect, variance analysis (ANOVA) and correlation between growth and physiological traits were performed using SPSS18.0 (IBM Corporation, New York, USA).

### DNA isolation and SSR analysis

Young leaves of each accession were collected for DNA isolation using a cetyltrimethyl ammonium bromide (CTAB) method [56]. A set of 90 published genome-wide SSR markers [21,57] mapped in 22 linkage groups in tall fescue were analyzed in all accessions (Additional file 1 Table S1). All forward primer sequence of markers were labeled with four fluorescent dyes of different colors [FAM (blue), HEX (green), TAMRA (yellow), and ROX (red)]. Each 10 μL PCR reaction in 96 microplates consisted of 1 × supplied *Taq*-buffer, 2.5 mM MgCl_2_, 200 μM dNTPs, 0.2 mM of each primer pair, 0.5 U of *Taq* DNA polymerase, and 30 ng of template DNA. PCR reaction was started at 95°C for 10 min; followed by 25 cycles of 50 s at 95°C, 50 s at 68°C with a decrease of 0.6°C in each consequent cycle, 60 s at 72°C; then ran for 15 cycles at 95°C for 50 s, 54°C for 50 s, 72°C for 60 s; and a final extension step 72°C for 10 min. All PCR reactions were used a touch-down program in a 96-well My Cycler thermal cycler (Bio-Rad Inc., Hercules, CA, USA). The PCR amplified fragments were separated by an ABI 3730 DNA Sequence (Applied Biosystems Inc., Foster City, CA, USA). Alleles were scored by GeneMarker 1.5 software (Soft Genetics, LLC, State College, PA, USA) and checked twice manually for accuracy. If more than one fragment were amplified by a primer in accession and appeared differently in other accessions, they were scored as different loci. For allohexaploid genome of *F. arundinacea*, band scores of SSR loci were entered into a binary matrix as presence (1) or absence (0) following Sara et al. [21]. All confirmed polymorphic alleles were applied for population structure and kinship analysis.

### Population structure and relative kinship

As a result of labeling all SSR markers as dominant in each genotype, no information on marker linkage could be obtained in population structure model. A Bayesian model-based clustering method carried out in STRUCTURE 2.0.1 software [58] was employed to determine population structure (Q) and division accessions into subpopulation. The basis of the clustering method is that it prevented admixture of correlated allele frequencies, therefore the allocation of individual genotype to K subpopulations is in such a way that Hardy-Weinberg and linkage equilibrium is valid within populations. The structure was run ten times by setting pre-defined k (the number of population groups) ranging from 1 to 15 using admixture models with 10,000 MCMC (Markov Chain Monte Carlo) replications and 10,000 burn-in time for each run. Population based on the maximum likelihood was determined by the probability of data likelihood *LnP(D)* in the output and an ad hoc statistic △K based on the second-order rate of change in *LnP(D)* between successive K values [58]. 15 independent runs were operated 100 000 iterations of each run after burn-in of 100 000 for a value of K setting from one to five. Then SPAGeDi software [59] was applied to evaluating relative pairwise kinship (K) by 90 SSR markers, and then the pairwise kinship matrix (100 × 100) was produced by the Loiselle coefficient [60]. All negative kinship values between the individuals were assigned to zero, according to Yu et al. [41].

### Model testing and association mapping

Based on the differences in the regime of heat treatment (density, duration time and matrix cultivated plant), the functional traits of heat tolerant in two trials were used for identifying association with SSR loci, respectively. Turfgrass quality, growth rate and leaf chlorophyll content in growth chamber trial were selected to perform marker-trait associations. Three models were used to access the effects of relative kinship (*K*) and population structure (*Q*) for marker-trait associations. The *Q* model was performed using general linear model (GLM). The *K* and *K + Q* models were performed using MLM in TASSEL 2.0.1 software [58]. The quantile- quantile plots of estimated –log_10_ (*P*) were drawn using the observed *P* values from SSR alleles-trait associations and the expected *P* values assuming that there was no associations identified between marker and trait. The significant threshold for marker-trait associations was set at *P <* 0.01.

## Additional files

Additional file 1:
**Supplementary Table S1.** List of the amplified information of polymorphism SSR markers. Additional file 2:
**Supplementary Table S2.** The values of membership probability for assigning tall fescue accessions to subpopulation when the k = 2. Additional file 3:
**Supplementary Table S3.** The associated marker alleles with five traits at two time points by *Q* model, *K* model and *Q + K* model in growth chambers trial (*P* < 0.01). Additional file 4:
**Supplementary Table S4.** The associated marker alleles with five functional traits at two sampling times by *Q + K* model in greenhouse trial and growth chambers trial, respectively (*P* < 0.01).

## Abbreviations

CHL: Chlorophyll content
ET: Evapotranspiration rate
FW: Fresh leave weight
GLM: General linear model
GR: Growth rate
GWS: Genome-wide association
MAS: Marker-assisted selection
MLM: Mixed linear model
MTA: Marker-trait association
SR: Survival rate
SSR: Simple sequence repeat
TQ: Turfgrass quality
